# Exploration and Consideration of the Medical Alliance Modes

**Published:** 2018-08

**Authors:** Ying CAI, Cheng WEN, Long TANG, Pufei LIU, Yan XU, Suxia HU, Miao WEI, Junhua CAO

**Affiliations:** Dept. of Nursing, Xuzhou Children’s Hospital, Xuzhou 221006, China

**Keywords:** Medical alliance, Mode, China

## Abstract

**Background:**

The distribution of medical resources in China has long been in an unbalanced state. Since 2009, the government has initiated the planning and construction of a sound grass-roots medical and health service system, increased investment in grass-roots medical and health institutions, but it has not received the expected results. In order to solve the problem, the medical consortium—with a full-featured, hierarchical and resource-sharing structure has been proposed.

**Methods:**

Overall, 1000 Electronic questionnaires about cognitive status of residents on medical alliance were randomly distributed in 50 community health service centers in 10 cities including Xuzhou, Nanjing, Hefei, Jinan, Zhengzhou, Changsha, Wuhan, Xi’an, Nanchang and Chengdu, China.

**Results:**

94.84% of the respondents responded they had heard about the construction of medical alliance, but they did not know the specific content. When asked about the preferred medical institution after illness, 93.50% participants preferred third-tier general hospital or specialist hospital. 62.58% of the respondents believe that the establishment of the medical alliance has not yet played a role and they are concerned that it cannot be effectively implemented. 62.27% of respondents were attracted by the convenience of medical alliance, 20.72% of respondents thought that they could easily get to famous doctors when they were in need, and 5.46% of respondents thought that there was no advantage in medical alliance.

**Conclusion:**

The establishment of the medical alliance is an effective means to promote the optimal allocation of medical and health resources. Government should perform its functions, and medical institutions and the whole society should all participate.

## Introduction

Medical consortium refers to the formation of a collaborative alliance or medical group with different types and levels of medical and health organizations in a certain area through the integration of vertical and horizontal resources, and a combination of medical information and sharing of responsibility and benefits. Medical consortium is also known as medical alliance (1). Patients can enjoy convenient and high-quality diagnosis and treatment services such as two-way referral between the primary care institutions and regional medical centers, mutual recognition of laboratory test results, expert-to-community consultation, and long-distance consultation in medical alliance (2). Establishment of medical alliance is an extension of the ideas and approaches for reform of public hospitals. With medical alliance, the overall efficiency of medical service organizations has increased, and the improvement of primary medical service capabilities and collaborative service functions has important implications (3).

The distribution of medical resources in our country has long been in an unbalanced state. This has led to the “tertiary hospitals squeezing the threshold, deserted house in grass-roots hospital” phenomenon of long-standing (4). Since 2009, the government has started to improve the planning and construction of grass-roots medical and health service system so as to solve the problem of “seeing a doctor hard and seeing a doctor expensive” during the medical reform. To solved the basic obstacles for patients to go to the community medical institutions. However, huge investment did not bring the expected results. A large number of equipment purchased by primary medical institutions is idle, resulting in the waste of medical resources (5). How to solve the increasingly difficult problem of getting medical treatment in current situation of insufficient total quality medical resources, unbalanced distribution of medical resources, and lack of irrational structure has become an important constraint for ensuring people’s health and deepening medical reform.

Integrating existing medical institutions at all levels and establishing a new type of medical institution with integrated functions, distinct levels and resources sharing -- Medical Alliance (6), to solve the increasingly difficult problem of seeing a doctor. The Ministry of Health issued a document encouraging the development of a “medical alliance” and urged the public hospitals to promote the improvement of health services in grass-roots medical institutions of grading treatment (7) was expressed on the National Health Work Conference held in January 2013. Carrying out the construction of medical alliance is an important step in deepening medical reform and system innovation, which is conducive to adjusting and optimizing the structure and layout of medical resources, and improving grass-roots service. The overall effectiveness of the medical service system, the better implementation of graded treatment and to meet the health needs of the masses. Through literature search, it has been found from the 90s of the last century that the medical resources of our country began to integrate and the practice of medical alliance began to emerge. In 2009, the new medical reform proposal put forward and encouraged public hospitals to explore the reform of operation mechanism and upgrade the capacity of grass-roots medical and health services (8). The end of 2009, Jiangsu Rehabilitation Medical Group was established (9); 2011, the first medical alliance of Shanghai “Ruijin - Luwan Medical Alliance” was born (10); 2013 Ministry of Health for the first time explicitly encourage established medical alliance, hospitals throughout the country gradually opened up and practice of conjoined construction of various forms of the medical institutions. According to the integrated form of medical institutions can be divided into loose type of technology and compact medical group.

At present, China’s loose model of medical alliance is more common, which maintain their autonomy and independence in operation and management. On the other hand, they are usually oriented toward short-term economic interests and are not conducive to long-term development.

The compact medical alliance is to unify management of people, wealth and property on the basis of ownership and assets integration, forming a real responsibility community (11). Taking Jiangsu Zhenjiang rehabilitation medical group as an example, the medical alliance is linked by assets to build a close medical alliance with Zhenjiang First People’s Hospital as the core, a horizontal joint 5 secondary hospital and a longitudinal 10 community health service institutions. The main contents include the implementation of constructing the integrated platform for optimizing the integration of internal resources, give economic subsidies and promotion opportunities to encourage the doctor go to grass-roots institution, give guidance for medical service (12). The successful case of sinking and integration of high-quality medical resources, implementation of grading treatment and is worth learning.

Both medical alliances have achieved some success in the development process. We found many problems that still need to be resolved.

## Methods

Overall, 1000 Electronic questionnaires about cognitive status of residents on medical alliance were randomly distributed in 50 community health service centers in 10 cities including Xuzhou, Nanjing, Hefei, Jinan, Zhengzhou, Chang-sha, Wuhan, Xi’an, Nanchang and Chengdu, China. Research Committee of the hospital approved the study.

## Results

A total of 970 valid questionnaires were retrieved. Residents’ perceptions are presented in Table 1.

Among those participants, 94.84% responded they had heard about the construction of medical alliance, but they did not know the specific content, 93.50% of participants preferred third-tier general hospital or specialist hospital, and 89.07% of respondents mentioned they would not return to the community hospital for treatment after the condition was relieved.

**Table 1:** Survey on the level of cognition of medical alliance among residents of ten cities including Xuzhou, Hefei, Anhui and Jinan (n=970)

***Items***	***Cases* (n)**	***Proportion* (%)**
**Do you know the establishment of the medical alliance?**		
I heard that but don’t know the content	920	94.84
Heard a lot	10	1.03
Never heard but would like to know	30	3.09
Never heard or cared	10	1.03
**Preferred type of hospital**		
Third-tier general hospital or specialist hospital	907	93.5
Community Hospital	33	3.4
County hospital or township hospital	30	3.09
**Whether the patient is willing to return to community hospitals after improvement**		
Not willing to go to community hospital	864	89.07
Willing to with improvements	40	4.12
Willing to if doctors strongly suggest	66	6.8
**How do you evaluate the establishment of the medical alliance?**		
Has not played a role and is concerned that it cannot be implemented effectively	607	62.58
Not yet working, but It will work.	135	13.92
Unclear	200	20.62
Already working to solve many problems	28	2.89
**Advantages of medical alliance**		
Treatment convenience	604	62.27
It’s easy to reach famous doctors	201	20.72
Enjoy good medical technology and have a high reimbursement rate	112	11.55
No advantage/advantage	53	5.46

62.58% of the respondents believe that the establishment of the medical alliance has not yet played a role and they are concerned and that it cannot be effectively implemented. 62.27% of respondents were attracted by the convenience of medical alliance, 20.72% thought that they could easily get to famous doctors when they were in need, and 5.46% believed that there was no advantage in medical alliance.

## Discussion

### Problems and analysis of medical alliance in China

The existing management system restricts the development of medical alliance. At present, the government has not yet established the uniform standards and norms for the management. However, China has long implemented a hospital-level management system. In the meantime, due to the lack of unified management, more emphasis is actually given to talents and technical assistance. Medical staff have different personnel establishment cause to cannot be achieved free flow.

The existing model of profit distribution hinders the development of medical alliance. Now the development of tertiary hospitals is more likely to be in the “super-hospital” mode. The goal of the medical alliance is to achieve grading treatment, divert ordinary patients to grass-root medical institutions, and will change tertiary hospital’s benefits. Therefore, under the condition of economic independence of the medical alliance, the expansion of tertiary hospitals has a direct impact on the implementation of grading treatment. From the treatment cycle of the disease, the main expenses of patients in tertiary hospitals are for the project inspection, surgery and drug treatment, subsequent and rehabilitation treatment are not much profit margin, resulting in a two-way referral transfer difficult (13).

“Slight illnesses in the community”, “rehabilitation back to the community” are difficult to achieve. Tertiary hospitals have attracted patients to seek medical treatment. The patients are not willing to choose grass-roots medical institutions (14). In addition, there is a common problem in our country for the grading treatment that “it is difficult to turn downwards” (15). In terms of patients, tertiary hospital inpatients also expect to receive continuous treatment in large hospitals, contradicting top-down referrals, resulting in a more difficult two-way referral.

The core and starting point of the development is resource sharing, the information is still in the construction stage.

Informationization of hospitals in China is still in construction phase, and construction of various information platforms is not perfect. Informationization process of units of medical alliance is not uniform and information systems are not the same. Since the primary medical institutions are restricted by the economic level, the level of informatization cannot keep up with third-tier general hospital, resulting in poor registration, referral, and mutual recognition platforms.

Medical insurance policy also has some problems. From the perspective of patients, the reimbursement rate of medical insurance in tertiary hospitals and community institutions is not very different. From the perspective of the hospital, the medical insurance has not yet achieved the unified settlement within the medical alliance. The over-payment of medical insurance in the region is serious and affects the hospital’s economic interests.

Supporting policies are not perfect; hinder the development of grass-roots institutions. On the one hand, the price adjustment for medical services in various places has not taken more consideration of grass-roots institutions. On the other hand, there is a large different between the drug list of the grass-roots institutions and tertiary hospitals influence the choice of patients (16).

### Suggestions for the development of medical alliance

The optimization of the management system should obtain more personnel and managerial autonomy, and achieve “separation of management and operation.” Drawing on the Integrated Delivery System (IDS) model in the US, the corporate governance structure of the president-in-charge system under the leadership of the board is established. Unified standard of diagnosis and treatment, discipline construction plan, and the performance appraisal system are gradually established to control the talent flow. Under the leadership of the tertiary hospital, we should improve the service ability of grass-roots institutions and the multi-point practice policy to promote grading treatment.

The benefit between tertiary hospitals and lower medical institutions is an important issue that hinders the development of medical alliances and the implementation of grading treatment. Only by establishing a reasonable profit-sharing mechanism can promote grading treatment. The government should give financial policy support in the process of promoting the development, help tertiary hospitals return to the nature of public welfare.

In process of establishing a close-linked medical alliance, the medical community should be gradually given management autonomy, personnel autonomy, and income distribution autonomy, which will help it optimize resource allocation and rationally distribute benefits through performance appraisal.

Only enhance the service capabilities of medical professionals in grass-roots medical institutions, the masses will tend to choose from the point of view of convenience. And we could achieve by following aspects. 1) Achieve the function of “community, help and teach” with the policies of “counterpart assistance”; 2) To improve the medical staff’s professional ability training in different units; 3) To retain professionals through the establishment of performance appraisal system; 4) Tertiary hospitals led by the medical alliance shall formulate routine disease diagnosis and treatment procedures in the region and enhance medical treatment. In addition, the establishment of a scientific, reasonable, simple and efficient “two-way referral” process can promote the patient to turn local hospitals.

The role of the Kaiser shows the important role of information communication in medical alliance. Through the support of financial policies and the help of tertiary hospitals, developed “Internet + medical services” and built an integrated information exchange platform to achieve the exchange and mutual recognition of medical records and examination results, health follow-up file sharing. At the same time, through the co-construction booking, registration, referral platform to achieve the all unit’s members of online booking referral, admissions mode. And also can realize the high-quality resource sharing among the member units enhance the service level of the grass-roots institution.

In the aspect of health insurance, we could establishment of a new medical insurance settlement method which the core hospital will uniform all costs and calculate proportion of the expenses incurred by patient for different grade hospitals (17). You can also use the leverage of insurance reimbursement ratio to guide patients to give priority to community institution treatment. In the medical service price adjustment, two-way research should be conducted in consideration of the reasonable interests of grass-roots institutions and the promotion of development. In patients with medication, through the establishment of pharmacies, improve the drug catalogue, to avoid the reasons for the drug due to the patient transfer.

This study summarizes the background, current status, and problems of China’s current medical alliance construction, and concludes that residents’ cognitive level on medical alliance needs to be improved. We analyzed the problems existing in the development of medical alliance and put forward suggestions. Government should perform its functions, and all medical institutions and the whole society shoul participate. Establishment of medical alliance is an extension of the ideas and approaches for reform of public hospitals.

## Conclusion

Establishment of medical alliance is of great significance to the improvement of the overall efficiency of medical service organizations, and the enhancement of basic medical service capabilities and collaborative service functions.

## Ethical considerations

Ethical issues (Including plagiarism, informed consent, misconduct, data fabrication and/or falsification, double publication and/or submission, redundancy, etc.) have been completely observed by the authors.
